# Prenatal Maternal Immune Activation with Lipopolysaccharide Accelerates the Developmental Acquisition of Neonatal Reflexes in Rat Offspring Without Affecting Maternal Care Behaviors

**DOI:** 10.3390/biom15030347

**Published:** 2025-02-27

**Authors:** Mary Beth Hall, Elise A. Lemanski, Jaclyn M. Schwarz

**Affiliations:** 1 Department of Psychological and Brain Sciences, University of Delaware, Newark, DE 19716, USA; lemanski@udel.edu; 2 Interdisciplinary Neuroscience Graduate Program, University of Delaware, Newark, DE 19716, USA

**Keywords:** maternal immune activation, neurodevelopmental disorders, lipopolysaccharide, neonatal reflex development, maternal care

## Abstract

Maternal immune activation (MIA)—infection with an immunogen during pregnancy—is linked to an increased risk of neurodevelopmental disorders (NDDs) in offspring. Both MIA and NDDs are associated with developmental delays in offsprings’ motor behavior. Therefore, the current study examined the effects of MIA on neonatal reflex development in male and female offspring. Sprague Dawley rats were administered lipopolysaccharide (LPS; 50 μg/mL/kg, i.p.) or saline on embryonic day (E)15 of gestation. The offspring were then tested daily from postnatal day (P)3–P21 to determine their neonatal reflex abilities. The maternal care behaviors of the dam were also quantified on P1–P5, P10, and P15. We found that, regardless of sex, the E15 LPS offspring were able to forelimb grasp, cliff avoid, and right with a correct posture at an earlier postnatal age than the E15 saline offspring did. The E15 LPS offspring also showed better performance of forelimb grasping, hindlimb grasping, righting with correct posture, and walking with correct posture than the E15 saline offspring did. There were no significant differences in maternal licking/grooming, arched-back nursing, non-arched-back nursing, or total nursing across the E15 groups. Overall, these findings suggest that MIA with LPS on E15 accelerates reflex development in offspring without affecting maternal care. This may be explained by the stress acceleration hypothesis, whereby early-life stress accelerates development to promote survival.

## 1. Introduction

Prenatal infection associated with maternal immune activation (MIA) has been linked to an increased risk of offspring being diagnosed with and/or experiencing symptoms of neurodevelopmental disorders (NDDs) [1,2,3,4]. Epidemiological studies in humans classify MIA as maternal infection with an immunogen (e.g., bacterial, viral, parasitic) during pregnancy [5]. In animals, MIA can be modeled via direct infection (e.g., with a virus, bacteria, or parasite) or via stimulation of the immune system in the absence of overt infection (e.g., with a viral or bacterial mimetic, an immune-related molecule, or an environmental stressor) [5,6,7,8]. Two common rodent models of MIA include administration of polyinosinic:polycytidylic acid (Poly I:C) or lipopolysaccharide (LPS), which are mimetics for viral and bacterial infections, respectively [7,8]. Rodent studies of MIA are essential to better understand how immune activation during gestation may causally affect the development of behavioral and neurobiological symptoms underlying NDDs.

According to the DSM-5 (*Diagnostic and Statistical Manual of Mental Disorders, 5th Edition*), NDDs are a group of disorders that produce functional impairments in one or several areas of development, such as language, motor, social, and learning skills [9]. The prevalence of NDDs amongst children aged 3–17 in the United States is ~17% [10], with boys more likely to be diagnosed than girls [11,12]; however, the etiology of these disorders is not well understood. NDD diagnoses in the United States include autism spectrum disorder (ASD), attention deficit/hyperactivity disorder (ADHD), intellectual disability (ID), and communication disorders. Many researchers also consider early-onset schizophrenia to fall into the NDD category because its etiology likely results from events that occur during early development [13,14,15,16]. Notably, these different NDD diagnoses have overlapping symptoms, including cognitive and learning disabilities, altered sleep and circadian patterns, metabolic/gastrointestinal issues, and delays in terms of behavioral reflexes and milestones during development [5,17,18]. There are additionally high rates of comorbidity within NDDs, and between NDDs and other psychiatric disorders [19], suggesting that there are similar causes (e.g., MIA) and neurobiological mechanisms underlying these disorders. Still, it is important to note that individuals may experience different symptoms (and/or symptom severity) within and across various NDDs.

### 1.1. Developmental Reflex Acquisition

MIA and NDDs are both associated with the delayed attainment of developmental milestones [17,18]. In human offspring, NDDs are associated with reductions in quality and delayed acquisition of motor reflexes across early postnatal development [20,21,22,23,24,25,26,27]. Children with ASD experience impairments in their gross and fine motor skills indicative of the delayed attainment of developmental milestones such as reflex acquisition (walking, righting, etc.) and grasping objects [20,21,22,23,24]. Similarly, delays in walking, standing, and sitting unsupported are associated with later schizophrenia diagnoses in offspring [25,26,27]. One potential explanation for such differences in the motor reflex abilities of children with NDDs may be due to hypotonia [22,28,29]; however, the exact cause is still unknown.

Whereas the human evidence described above seems to agree that NDDs are associated with delayed motor development, rodent models of MIA provide mixed results [30,31,32,33,34]. A mouse model of MIA with Poly I:C on E12.5 caused delayed righting ability (flipping from a supine to prone position) in male offspring and delayed eye opening in female offspring [30]. Similarly, MIA with Poly I:C, but not LPS, from E15 to E17 produced delays in the righting and grasping abilities in mouse offspring [31]. However, there are also studies that report no differences in righting, grasping, eye opening, or cliff avoidance reflexes following MIA with Poly I:C [32] and with LPS [33,34]. On the other hand, there is also evidence to suggest that MIA with LPS (50 µg/kg) from E15 to E16 produces *accelerated* abilities in terms of grip strength and geotaxis yet causes offspring to take longer to successfully cliff avoid [33]. Thus, it is unclear exactly how MIA impacts reflex acquisition across neonatal development in rats and how such outcomes may be related to early motor symptoms of NDDs.

### 1.2. Maternal Care Behavior

In rodents, the interactions between the dam and the litter are known to be crucial factors for postnatal development. Variations in maternal licking/grooming (LG)—the dam licks/grooms the pup’s body and anogenital area [35]—and arched-back nursing (ABN)—a form of active nursing style where the dam holds herself up over the pups [36]—can have lasting effects on offsprings’ behavior and gene expression [37]. Other, less active nursing styles that are commonly observed in dams include blanket nursing and passive nursing [35]. Although rat dams exhibit natural variations in the levels of maternal care behaviors toward offspring [37], it is imperative to consider whether MIA systematically affects this natural variation across groups. Rodent models report mixed results regarding the effects of MIA on maternal behaviors. Some studies report that MIA with LPS (50 µg/kg) on E12 or E16 [34] and with Poly I:C [32] produces no differences in maternal care behaviors such as LG, ABN, and non-ABN. However, other studies report that MIA with LPS (100 µg/kg) on E9.5 [38] and E18 [39] causes quicker retrieval of rat pups back to the nest. On the other hand, a higher dose of LPS (250 µg/kg) on E21 delays pup retrieval and decreases maternal crouching behaviors but does not affect LG [40]. Additionally, MIA with Poly I:C on E12.5 in mice reduces LG and increases nest building behavior but does not affect overall nursing behaviors [41]. Overall, there are inconsistent findings pertaining to how MIA affects maternal care behaviors toward pups and contributes to long-lasting biological and behavioral phenotypes in offspring when there are differences in care.

### 1.3. Investigating the Effect of MIA on Neonatal Reflex Development in Offspring and Maternal Care Behaviors

The goal of the current study was to investigate how MIA, via prenatal exposure to LPS, in rats affects the emergence of reflex behaviors across neonatal development. Additionally, we examined interactions between the dams and litters across the neonatal period to exclude the possibility that maternal care, which is highly important for offspring behavioral and neurobiological development [35,37], impacted reflex development in offspring. We utilized a dosage of 50 µg/mL/kg of LPS on embryonic day 15 of rat gestation, which is equivalent to the second trimester in humans; in rats, the third trimester equivalent occurs postnatally [42]. In humans, MIA with bacterial infection during the second/third trimester is associated with an increased ASD risk and during the first trimester is associated with an increased schizophrenia risk [5]. Thus, the results of this study will allow us to better understand the effects of MIA on developmental reflex acquisition in male and female rat offspring, and how it may relate to the behavioral symptoms of NDDs.

Based on the epidemiological evidence that NDDs are associated with motor development delays [20,21,22,23,24,25,26,27], and that MIA is a risk factor for NDDs [1,2,3,4], we hypothesized that neonatal offspring prenatally exposed to LPS would exhibit delays in the onset of behavioral reflex milestones. Furthermore, since males are more likely than females to be diagnosed with NDDs [43], we also hypothesized that these developmental delays may be more evident in male than female offspring. Contrary to these hypotheses, we found that prenatal LPS accelerated neonatal reflex development, regardless of offspring sex. Finally, in line with previous MIA models of LPS, we predicted that there would not be any differences in maternal care behaviors such as LG and ABN between MIA exposure groups. In support of this hypothesis, we report that MIA did not affect these maternal care behaviors across the postnatal period.

## 2. Materials and Methods

### 2.1. Experimental Subjects

Seventy-six (38 male and 38 female offspring) Sprague Dawley rats (*Rattus norvegicus*) were derived from 19 litters (9 saline and 10 LPS dams) bred at the University of Delaware (Newark, DE, USA). Four offspring per litter (2 males and 2 females) were included in each experimental group (36 saline and 40 LPS offspring), and their behavioral reflex scores were averaged for *n* = 1 score per sex/litter/group. Adult male and female rats were ordered from Charles River Laboratories (Wilmington, MA, USA) and allowed at least one week of acclimation before breeding at the University of Delaware. Male and female pairs were housed together for no more than five days. Each morning, the cages were checked for the presence of a sperm plug, which was designated as embryonic day (E)1.

**Figure 1:**
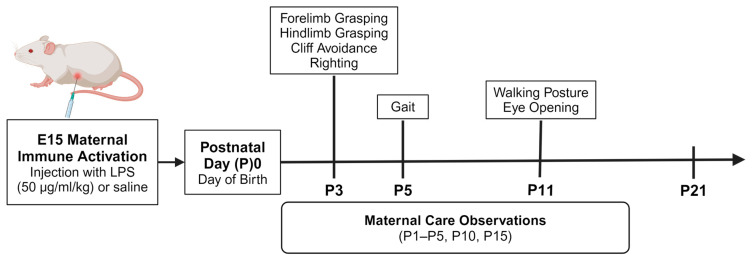
Experimental design. Male and female offspring (*n* = 1 pup per sex/litter/group for statistical analyses) underwent neonatal reflex testing daily from P3 to P21 (*n* = 18 saline pups; *n* = 20 LPS pups). The specific type of reflex test and the day that each test began are indicated in the figure above. The litters were also observed for maternal care behaviors on P1–P5, P10, and P15 (*n* = 9 saline dams; *n* = 10 LPS dams) to determine if there were group differences in maternal care at both the early (P1–P5) and later (P10, P15) neonatal stages. The maternal care behaviors were scored for instances of maternal licking/grooming of pups, arched-back nursing, non-arched-back nursing, and total nursing.

**Figure 2:**
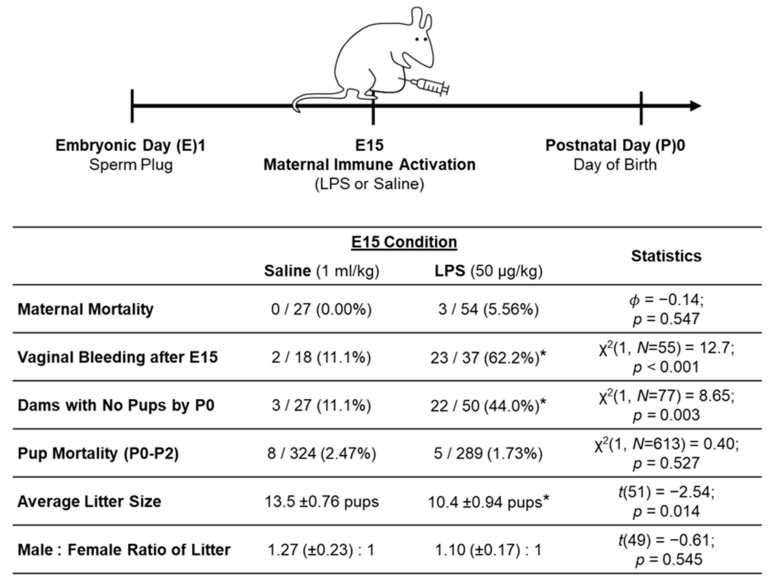
Schwarz Lab maternal immune activation (MIA) model. In our MIA model, which was developed across multiple experiments in our lab, LPS injection on E15 resulted in 3 maternal deaths and caused more dams to resorb the litter by P0, to experience vaginal bleeding post-injection, and to have smaller litter sizes than saline dams. * significantly different from saline (*p* < 0.05).

After breeding, the females were housed individually and provided a red tunnel, popsicle stick, cotton square, and crinkle paper for nesting materials. Females were weighed on E9 to determine a 10% increase in body weight as secondary confirmation of pregnancy and weighed again on E13. Cage changes occurred on E19, E13, and E21 so as not to overlap with the MIA injection on E15 and birth on E23, respectively. After birth, the dams and pups were left undisturbed until the experimental procedures began; the experimental design is summarized in Figure 1. Litters were not culled; see Figure 2 below for the mean ± SEM of litter sizes per E15 condition. The neonatal cages were provided with a red tunnel, crinkle paper, and cotton square. The sex of the offspring was established by assessing their anogenital distance on P2. After birth, cage changes occurred on P8, P13, and P18 so as not to interfere with the maternal care observations on P1–P5, P10 and P15, and to allow time for the dam to rebuild her nest prior to those observations.

The rats were housed in clear, polyethylene cages (45 cm × 20.5 cm × 24 cm) and maintained under standard laboratory conditions (22 °C). The rats were provided with standard laboratory food and tap water ad libitum. The colony room was kept on a 12:12 h light–dark cycle, with lights on at 7:00 a.m. The experimental procedures were performed during the light portion of the cycle. Sentinel rats were housed in the colony room and periodically examined for the presence of common rodent diseases; all the tests came back negative. All the experiments were approved by the University of Delaware Institutional Animal Care and Use Committee.

### 2.2. Maternal Immune Activation

#### 2.2.1. Lipopolysaccharide Preparation

Lipopolysaccharide derived from *Escherichia coli* O111:B4 (Sigma, St. Louis, MO, USA; Cat No. L2630-25MG) was reconstituted in 10 mL of saline (Dulbecco’s phosphate-buffered saline; Fisher, Manassas, VA, USA; Cat No. MT21030CV) to achieve a stock concentration of 2500 µg/mL, that was then stored at −80 °C in 100 µL aliquots. On the day of injection, LPS aliquots were thawed and further diluted with saline to achieve a working concentration of 50 µg/mL.

#### 2.2.2. MIA Injection on E15

On embryonic day (E)15 of gestation, prior to 12:00 p.m., the female rats were weighed and randomly selected to be injected with lipopolysaccharide (LPS; 50 µg/mL/kg, i.p.) or its vehicle, sterile saline (1 mg/kg), using a 26 G needle (Fisher, Nazareth, PA, USA; Cat No. 14-826-10). In a vacant procedure room, the dams were restrained using a towel wrap and held with their head beneath their feet in order to create space for injection into the intraperitoneal cavity; the needle was aspirated prior to injection to prevent injection into an organ or the bloodstream. The experimenters were not blinded to the E15 condition at the time of injection. Post-injection, the rats were weighed daily and monitored for vaginal bleeding (see Figure 2 for statistics) and other sickness behaviors until they gave birth, which was designated as postnatal day (P)0. Dams with substantial vaginal bleeding, or that lost more than 20% of their body weight, were euthanized.

#### 2.2.3. MIA Model Outcomes

See Table S1 in the Supplementary Materials for the recommended reporting guidelines regarding our MIA model [44]. In our original model of MIA, we injected 5 dams with 100 µg/mL/kg of lipopolysaccharide on E15. Researchers have reported using doses of LPS ranging from 25 μg/kg to over 1 mg/kg to produce rat models of MIA, and 100 μg/kg is the most common dose reported [7,8]. However, after using the 100 µg/kg dose, we observed significant vaginal bleeding and weight loss in 2 of 5 rats (40%) post-injection. We therefore decided to reduce the dose to 50 µg/mL/kg in the current experiment.

The lower dose of LPS (50 µg/kg) used in our lab and the current study still elicited differences between LPS and saline dams and litters; see Figure 2 for statistics. (Note: the statistics for our MIA model are derived from litters that were generated both for the current experiment and for additional experiments in our lab.) E15 LPS caused 3 maternal deaths (5.56%; that occurred by E16) and caused 62.2% of dams to have vaginal bleeding post-injection (between E16 and E18). LPS injection on E15 also caused 44.0% of dams to resorb the litter (no pups were born to these dams). The LPS dams that did go on to give birth had significantly smaller litter sizes than the saline dams. These findings are in line with previous MIA research that reports Poly I:C and LPS (administered across various gestational ages) to cause increased litter resorption and reduced size of litters born [30,45,46,47]. The implications of these MIA effects on the dams and pups are considered below in the discussion. Finally, we found no differences across the E15 conditions in the number of postnatal pup deaths from P0 to P2 or in the male-to-female ratio of the litters. The offsprings’ postnatal weights were also not significantly different across the E15 conditions (see Results section).

**Figure 3:**
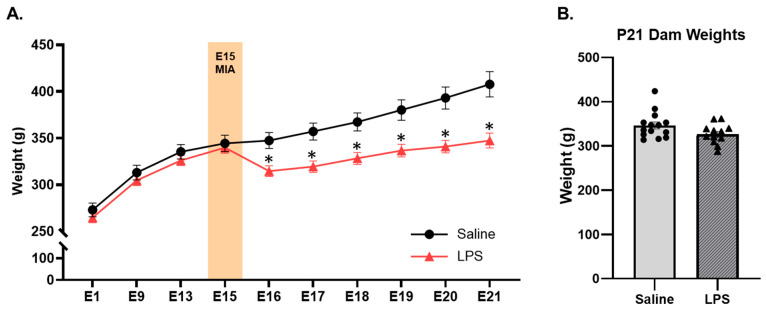
Dam weights from E1 to E21 and at P21. (**A**) Dams that received LPS on E15 weighed less post-injection (from P16–P21) than those that received saline. (**A**) Dams did not differ in their weights prior to injection on E15 (*n* 18–37 dams per group) or (**B**) following parturition at the time of weaning on P21 (*n* = 12–14 dams per group). * pairwise comparison; significantly different than saline (*p* < 0.05).

Furthermore, there were significant differences in weight between the E15 LPS and saline dams depending on their gestational age [*F*(9, 477) = 16.3, *p* = 0.001, η_p_^2^ = 0.235]; see Figure 3. Pairwise comparisons revealed that there were no differences in weight between the groups prior to E15 MIA injection. However, post-injection (from E16 to E21), the LPS dams weighed significantly less than the saline dams (~10–15% difference; *p*s < 0.05); this analysis included dams that did not have pups by P0 but excluded dams for maternal mortality. These findings align with a previous mouse study that reported a decrease in the food intake and body weight of pregnant dams following injections with LPS but not Poly I:C [31]. However, we did not measure food intake in the current study and are therefore unable to determine if this was the cause of the weight loss in our LPS dams. This weight difference between groups may also be due to partial or full reabsorption of the fetuses during gestation, as the LPS dams had significantly fewer successful births by P0 and gave birth to smaller litters than the saline dams did (see Figure 2). Notably, in the dams that successfully gave birth to pups by P0, there were no longer any differences across the E15 conditions in the dam weight at the time of weaning on P21 [*t*(24) = 1.93, *p* = 0.066].

### 2.3. Neonatal Reflex Behaviors

From P3 to P21, 2 male and 2 female pups (scores were averaged so that *n* = 1 per sex/litter in each experimental group to minimize possible litter effects) were randomly selected from each litter and observed daily to determine the ontogeny of neonatal reflex behaviors. The same 4 offspring from each litter were used throughout the entirety of the developmental testing. The offspring that underwent reflex testing were removed from the dam for no more than 30 min each day, were kept on a heating pad to maintain a stable body temperature, and were weighed daily at the time of testing. The pups that were not tested for reflex development remained with the dam during the testing period. The experimenters were blinded to the E15 condition at the time of testing.

Each behavioral milestone listed below was measured daily between 8:00 a.m. and 12:30 p.m. in a vacant procedure room, beginning at least one day prior to the typical onset of the behavior in rats [48]. The developmental reflex measures were adapted from Nguyen and colleagues [48] and are described below. Each reflex was deemed to be acquired when the pup could complete the task successfully for two days in a row, without regressing in their performance score on later days of testing. The second day of successful performance was considered the day of acquisition of the behavior. In between each litter tested, all the surfaces were wiped clean with Quatricide solution.

**Forelimb and hindlimb grasping.** From P3 to P21, an unfolded paperclip was placed against the pad of each forelimb and hindlimb with light pressure. The pups were observed to determine if they could grasp the paperclip (flexion of all the digits around the rod) with each forepaw and with each hindpaw. Scoring: 0 for no grasping; 1 for grasping by one paw; 2 for grasping by both paws.

**Cliff avoidance.** From P3 to P21, the pups were placed at the edge of a flat table with the lower half of their forelimbs and forepaws hanging over the edge. A blue ice bucket lined with a soft towel was held underneath the table to catch the pup in case they fell. The pups were observed to determine if they could turn away from the edge of the “cliff”. Scoring: 0 for no movement or falling off the edge; 1 for movement away from the edge but limbs were still hanging off; 2 for complete movement away from the edge.

**Righting.** From P3 to P21, the pups were held with their back on a flat table and all four paws upright. Once the pup was released, righting was deemed complete when the pup was able to roll over onto its stomach within 15 s. Righting with correct posture was considered to occur when the pup could roll over onto all four paws within 15 s and its stomach was not touching the table. Scoring: 0 for still lying on their back at the end of 15 s; 1 for righting within 15 s but with their stomach still touching the table; 2 for righting with correct posture within 15 s.

**Gait.** From P5 to P21, the pups were placed in the center of a 15 cm diameter circle outlined with a permanent marker on a plastic clipboard. The pups were observed to determine if they could move both forepaws outside of the circle within 30 s. Scoring: 0 for unable to move any forepaws outside the circle within 30 s; 1 for ability to move one forepaw outside the circle within 30 s; 2 for ability to move both forepaws outside the circle within 30 s.

**Walking posture.** From P11 to P21, the pups were placed on a flat table and observed to determine if they could walk with a mature posture, i.e., without their stomach dragging on the table and with their legs positioned underneath their body. An immature posture was considered to occur when the stomach dragged on the table, or the legs were splayed while moving. Scoring: 0 for no movement; 1 for immature posture when moving; 2 for mature posture when moving.

**Eye opening.** From P11 to P21, the pups were observed to determine when both eyelids were fully open. Scoring: 0 for no visible eyes; 1 for one visible eye (left or right) that was either cracked or fully open; 2 for two visible eyes but both were not fully open; 3 for two visible eyes that were both fully open.

### 2.4. Maternal Care Observations

Following birth, the litters were recorded (using UniFi^®^ Video software 3.10.13; Ubiquiti Inc., New York City, NY, USA) in their colony room with cameras (UniFi^®^ UVC G3; Ubiquiti Inc., New York City, NY, USA) positioned perpendicular to the home cages to capture their length. The recordings occurred for 30 min at 7:00 a.m. and 30 min at 3:00 p.m. on P1, P2, P3, P4, P5, P10, and P15. These times of day were chosen for the observations because nursing behaviors in rats have been found to occur more frequently during the light phase of the light–dark cycle [37]. The litters were observed from P1 to P5 to determine the maternal care behaviors for two days before (P1–P2) and two days after (P4–P5) neonatal reflex testing began on P3. The dams were additionally observed on P10 and P15 to determine whether differences in maternal care behaviors between groups emerged later during the neonatal period. Cage changes occurred 2–3 days prior to the recordings in order to minimize the potential exogenous effects on the dam’s interactions with the litter. The maternal behaviors of the dams were scored from the recordings (playback speed = recording speed) at each minute (totaling 62 observations) by a researcher blinded to the E15 condition. In cases where the dam’s behavior was ambiguous, the observers watched ±10 s of the video (relative to each minute mark) in order to contextualize and identify the behavior.

The videos were scored to determine instances of licking/grooming (LG) of pups and of arched-back nursing (ABN). ABN was defined as the dam nursing at least one pup while supporting herself with extended limbs, creating an “arched” look over the pup(s). LG and ABN actions are the most common maternal behaviors displayed after birth [37,49], often occur concurrently, and are important for offspring development and survival [36,50]. Instances of passive and blanket nursing were also scored to identify any differences in non-arched-back nursing (non-ABN). Blanket nursing was defined as the dam nursing at least one pup while not supporting herself, e.g., laying on the pup(s). Passive nursing was defined as the dam nursing at least one pup while laying on her side/back or if she was engaging in non-pup-directed behaviors such as eating, drinking, etc. Finally, the total amount of nursing (sum of both ABN and non-ABN behaviors) that occurred during our observations was calculated to determine if there were overall differences in the frequency of any nursing behavior across groups.

### 2.5. Statistical Analyses

Data were analyzed using the statistical software programs SPSS 29.0.2.0 (IBM, Armonk, NY, USA) and GraphPad Prism 10.4.0 (Dotmatics, Boston, MA, USA). The sample sizes were determined based on previous studies conducted in our lab. The researchers were not blinded to the experimental conditions at the time of data analysis.

For the neonatal reflex behaviors, there were 36 saline pups (18 male and 18 female) whose scores were averaged for *n* = 9 pups per sex for the statistical analyses, and there were 40 LPS pups (20 male and 20 female) whose scores were averaged for *n* = 10 pups per sex for the statistical analyses. Two-way ANOVAs with sex (male vs. female) and E15 condition (saline vs. LPS) as factors were performed to detect differences in the acquisition of each neonatal reflex behavior. Greenhouse–Geisser correction was used for analyses where the assumption of sphericity was violated. For data that violated Levene’s test of the equality of error variances of the mean, a Mann–Whitney U test was performed instead. Grubb’s outlier test was performed to identify outliers in each group and no more than one outlier per group was removed. Outliers were removed from the following groups: **forelimb grasping:** 1 female LPS; **righting with immature posture:** 1 female saline, 1 male saline; **righting with correct posture:** 1 female LPS.

Furthermore, a repeated-measure ANOVA with sex (male vs. female) and E15 condition (saline vs. LPS) as the between-subjects factors and postnatal day (P) as the within-subjects factor was used to analyze the differences in offspring weights and in the performance of neonatal reflex behaviors across development. There were no significant sex differences in the results of the repeated-measure ANOVA (all *p*s > 0.05), so the neonatal reflex development data were reanalyzed with the scores collapsed across sex. Sphericity was not assumed and therefore Greenhouse–Geisser correction was applied for analyses in which sphericity was violated. Pairwise comparisons with Bonferroni corrections were performed to further examine any significant interactions.

There were 9 saline litters and 10 LPS litters in the maternal care experiment. For the maternal care analyses, dams were excluded from the dataset if the observer was unable to identify care behaviors during the video due to an obstructed view of the dam and/or litter (e.g., the dam and/or nest were hidden behind the tunnel in the cage). The number of dams excluded from each group on each postnatal day were as follows: **P1:** 3 saline, 1 LPS; **P2:** 2 saline, 2 LPS; **P3:** 0 saline, 2 LPS; **P4:** 0 saline, 1 LPS; **P5:** 2 saline, 0 LPS; **P10:** 1 saline, 0 LPS; **P15:** 0 saline, 1 LPS. To account for these missing data, a mixed-effects model with the E15 condition (saline vs. LPS) as the between-subjects factor and the postnatal day (P) as the within-subjects factor was used to determine the differences in maternal care behaviors (ABN, LG, non-ABN, and total nursing) instead of a repeated measures ANOVA. The mixed-effects model (GraphPad Prism 10.4.0; Dotmatics, Boston, MA, USA) uses a compound symmetry covariance matrix and is fit using the restricted maximum likelihood (REML). In the presence of missing values (missing completely at random), the results can be interpreted like a repeated measures ANOVA. Sphericity was not assumed and therefore Greenhouse–Geisser correction was applied for analyses in which sphericity was violated. Additionally, to further quantify the differences in maternal care across E15 conditions at each postnatal day, the Holm–Šídák method was used to perform multiple unpaired *t*-tests for each postnatal day. Unpaired *t*-tests with Welch’s correction were applied for the ABN, non-ABN, and total nursing analyses, as homogeneity of variance was violated for the following datasets: P2 ABN, P3 non-ABN, P2 total nursing, and P4 total nursing. Finally, for both the mixed-effects analysis and multiple unpaired *t*-tests, Grubb’s outlier test was performed to identify outliers in each group and no more than one outlier per group was removed. Outliers were removed as follows: **P3 ABN:** 1 LPS; **P2 non-ABN:** 1 LPS; **P3 non-ABN:** 1 saline; **P4 non-ABN:** 1 LPS; **P2 total nursing:** 1 LPS; **P3 total nursing:** 1 LPS.

## 3. Results

### 3.1. Offspring Weights

**Figure 4:**
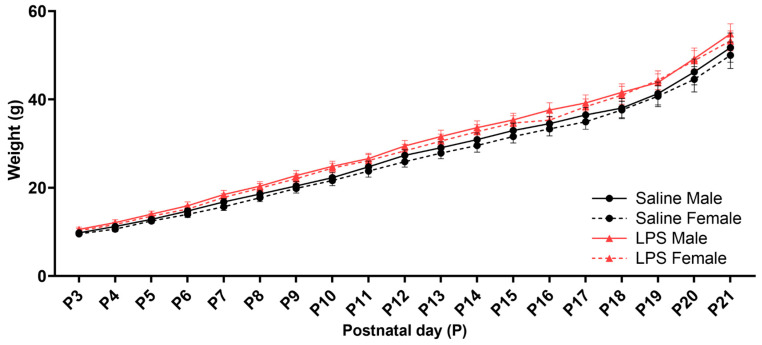
Offspring weights from P3 to P21. There were no significant differences in the postnatal weights across the E15 conditions or across sex in the offspring that were tested for neonatal reflex acquisition. *n* = 9–10 rats per sex/group. Error bars represent ±SEM.

The male and female offspring from each litter were weighed daily during the neonatal reflex acquisition testing. There was a significant effect of postnatal day [*F*(1.17, 39.8) = 961, *p* < 0.001, η_p_^2^ = 0.966], such that offspring weight increased over time regardless of sex and E15 condition; see Figure 4. There were no significant differences in the offspring weights as a function of sex [*F*(1, 34) = 0.402, *p* = 0.531], E15 condition [*F*(1, 34) = 3.05, *p* = 0.090], or their interaction [*F*(1, 34) = 0.009, *p* = 0.924]. There were no significant interactions between postnatal day and sex [*F*(18, 612) = 0.309, *p* = 0.998] or postnatal day and E15 condition [*F*(18, 612) = 1.14, *p* = 0.312]. There was also no significant interaction between postnatal day, sex, and E15 condition [*F*(18, 612) = 0.104, *p* > 0.999].

### 3.2. Neonatal Reflex Acquisition

**Figure 5:**
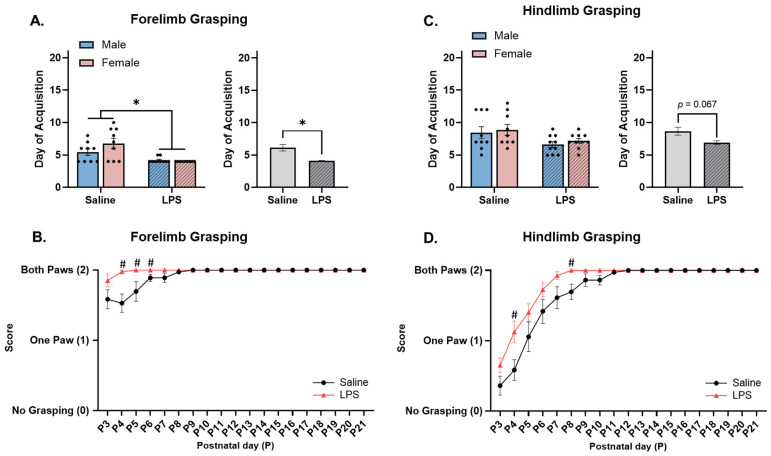
Forelimb and hindlimb grasping. (**A**) Offspring exposed to E15 LPS acquired forelimb grasping earlier in age than E15 saline offspring. (**B**) E15 LPS offspring performed better at forelimb grasping than E15 saline offspring from P4 to P6. (**C**) There were no differences in the acquisition of hindlimb grasping ability across the E15 conditions; however, (**D**) E15 LPS offspring performed better at hindlimb grasping than E15 saline offspring on P4 and P8. * main effect of E15 condition (*p* < 0.05). ^#^ pairwise comparison; significantly different than saline (*p* < 0.05). *n* = 9–10 rats per sex/group. Error bars represent ±SEM.

**Forelimb grasping.** The offspring were tested from P3 to P21 to determine their ability to grasp a paperclip rod with their forepaws. Levene’s test revealed a violation of homogeneity between the groups [*F*(3, 33) = 20.4, *p* < 0.001]; therefore, a nonparametric test was run. Since the two-way ANOVA revealed no significant differences across sex [*F*(1, 33) = 1.50, *p* = 0.230], the data were collapsed across sex. The Mann–Whitney test revealed a significant effect of E15 condition [*U* = 73.5, *p* = 0.002, *r* = 0.572], such that the offspring exposed to E15 LPS were able to successfully forelimb grasp at an earlier age (*M* = 4.11, *SEM* = 0.072) as compared to those exposed to E15 saline (*M* = 6.11, *SEM* = 0.484); see Figure 5A.

The repeated measures ANOVA revealed a significant effect of postnatal day [*F*(2.18, 78.6) = 9.54, *p* < 0.001, η_p_^2^ = 0.209], E15 condition [*F*(1, 36) = 10.1, *p* = 0.003, η_p_^2^ = 0.219], and their interaction [F(18, 648) = 5.30, *p* < 0.001, η_p_^2^ = 0.128] on forelimb grasping ability. Pairwise comparisons revealed that the E15 LPS offspring performed better at forelimb grasping on P4 (*p* = 0.003), P5 (*p* = 0.045), and P6 (*p* = 0.042) as compared to the E15 saline offspring; see Figure 5B.

**Hindlimb grasping.** The offspring were tested from P3 to P21 to determine their ability to grasp a paperclip rod with their hindpaws. Levene’s test revealed a violation of homogeneity between the groups [*F*(3, 34) = 5.39, *p* = 0.004]; therefore, a nonparametric test was run. Since the two-way ANOVA revealed no significant differences across sex [*F*(1, 34) = 0.614, *p* = 0.439], the data were collapsed across sex. The Mann–Whitney test revealed no significant effect of E15 condition on acquisition of hindlimb grasping [*U* = 117, *p* = 0.067]; see Figure 5C.

The repeated measures ANOVA revealed a significant effect of postnatal day [*F*(2.07, 74.3) = 81.1, *p* < 0.001, η_p_^2^ = 0.693], E15 condition [*F*(1, 36) = 6.57, *p* = 0.015, η_p_^2^ = 0.154], and their interaction [*F*(18, 648) = 3.07, *p* < 0.001, η_p_^2^ = 0.079] on hindlimb grasping ability. Pairwise comparisons revealed that the E15 LPS offspring performed better at hindlimb grasping on P4 (*p* = 0.015) and P8 (*p* = 0.012) than the E15 saline offspring did; see Figure 5D.

**Figure 6:**
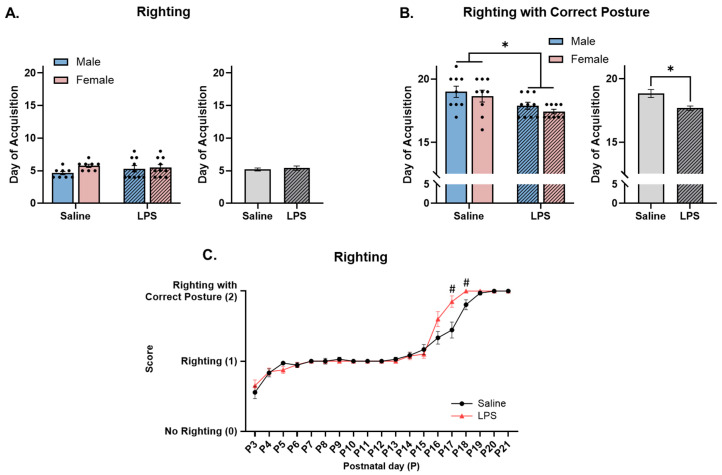
Righting. (**A**,**C**) There were no differences in the ability to right with an immature posture across E15 conditions. (**B**) Offspring exposed to E15 LPS acquired righting with a correct posture earlier in age than E15 saline offspring. (**C**) E15 LPS offspring performed better at righting with a correct posture than E15 saline offspring from P17 to P18. * main effect of E15 condition (*p* < 0.05). ^#^ pairwise comparison; significantly different than saline (*p* < 0.05). *n* = 8–10 rats per sex/group. Error bars represent ±SEM.

**Righting with immature posture.** The offspring were tested from P3 to P21 to determine their righting ability, i.e., if they could flip from a supine to prone position. Levene’s test revealed a violation of homogeneity between the groups [*F*(3, 32) = 4.90, *p* = 0.006]; therefore, a nonparametric test was run. Since the two-way ANOVA revealed no significant differences across sex (*F*(1, 32) = 2.60, *p* = 0.117), the data were collapsed across sex. The Mann–Whitney test revealed no effect of E15 condition on acquisition of righting across time [*U* = 157, *p* = 0.937]; see Figure 6A.

**Righting with correct posture.** The offspring were tested from P3 to P21 to determine their ability to right with a correct posture, i.e., if they could flip from a supine to prone position with their legs under their body and stomach off the table. Levene’s test revealed a violation of homogeneity between the groups [*F*(3, 33) = 2.93, *p* = 0.048]; therefore, a nonparametric test was run. Since the two-way ANOVA revealed no significant differences across sex [*F*(1, 33) = 1.21, *p* = 0.279], the data were collapsed across sex. The Mann–Whitney test revealed a significant effect of E15 condition [*U* = 80.0, *p* = 0.005, *r* = 0.470], such that the offspring exposed to E15 LPS successfully acquired righting with a correct posture at an earlier age (*M* = 17.5, *SEM* = 0.221) than those exposed to E15 saline (*M* = 18.8, *SEM* = 0.316); see Figure 6B.

**Righting ability across postnatal day.** The repeated measures ANOVA revealed no significant difference in righting ability as a function of E15 condition [*F*(1, 36) = 2.83, *p* = 0.101]. There was a significant effect of postnatal day [*F*(4.17, 150) = 187, *p* < 0.001, η_p_^2^ = 0.839] and a significant interaction between postnatal day and E15 condition [*F*(18, 648) = 3.52, *p* < 0.001, η_p_^2^ = 0.089] on righting ability. Pairwise comparisons revealed that the E15 LPS offspring performed better at righting—more specifically, righting with a correct posture—on P17 (*p* = 0.007) and P18 (*p* = 0.015) than the E15 saline offspring did; see Figure 6C.

**Figure 7:**
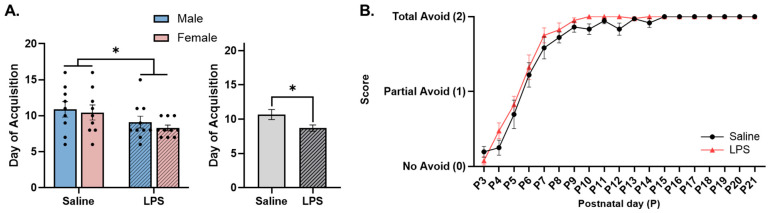
Cliff avoidance. (**A**) Offspring exposed to E15 LPS acquired cliff avoidance earlier in age than E15 saline offspring. (**B**) There was no difference in cliff avoidance performance between E15 offspring conditions. * main effect of E15 condition (*p* < 0.05). *n* = 9–10 rats per sex/group. Error bars represent ±SEM.

**Cliff avoidance.** The offspring were tested from P3 to P21 to determine their ability to turn away from the edge of the table, i.e., avoid the “cliff”. There were no significant differences in the acquisition of cliff avoidance as a function of sex [*F*(1, 34) = 0.519, *p* = 0.476] or the interaction between sex and E15 condition [*F*(1, 34) = 0.042, *p* = 0.838]. There was a significant effect of E15 condition [*F*(1, 34) = 5.19, *p* = 0.029, η_p_^2^ = 0.132], such that the offspring exposed to E15 LPS were able to successfully avoid the “cliff” at an earlier age (*M* = 8.63, *SEM* = 0.473) than those exposed to E15 saline (*M* = 10.67, *SEM* = 0.737); see Figure 7A.

The repeated measures ANOVA revealed a significant effect of postnatal day [*F*(3.55, 128) = 144, *p* < 0.001, η_p_^2^ = 0.800] on cliff avoidance ability, such that the offspring performed better across postnatal age regardless of the E15 condition. There was no significant effect of E15 condition [*F*(1, 36) = 3.40, *p* = 0.074] or the interaction between postnatal day and E15 condition [*F*(18, 648) = 0.759, *p* = 0.749]; see Figure 7B.

**Figure 8:**
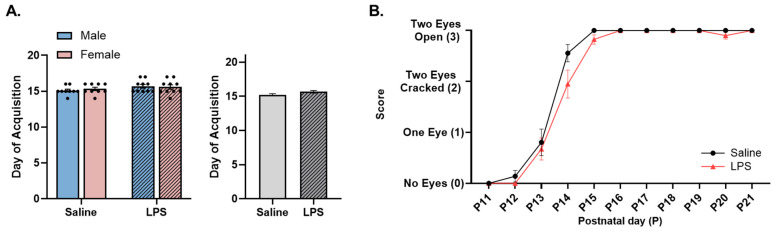
Eye opening. (**A**) There were no differences in the day of acquisition of eye opening across the E15 conditions. (**B**) There was no difference in eye opening ability between E15 offspring conditions. *n* = 9–10 rats per sex/group. Error bars represent ±SEM.

**Eye opening.** The offspring were tested from P11 to P21 to determine their ability to fully open both eyes. There were no significant differences in the age at which the offsprings’ eyes were fully open as a function of sex [*F*(1, 34) = 0.056, *p* = 0.814], E15 condition [*F*(1, 34) = 2.76, *p* = 0.106], or their interaction [*F*(1, 34) = 0.391, *p* = 0.536]; see Figure 8A.

The repeated measures ANOVA revealed a significant effect of postnatal day [*F*(2.00, 72.1) = 311, *p* < 0.001, η_p_^2^ = 0.896], such that the offspring were able to more fully open their eyes across time, regardless of their E15 condition. There was no significant effect of E15 condition [*F*(1, 36) = 2.68, *p* = 0.110] or the interaction between the postnatal day and E15 condition [*F*(10, 360) = 1.59, *p* = 0.106]; see Figure 8B.

**Figure 9:**
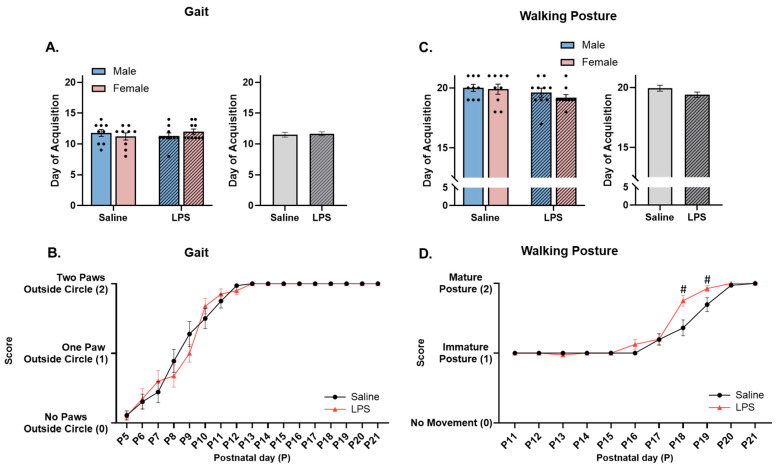
Acquisition of gait and walking posture. (**A**,**C**) There were no differences in the acquisition of gait or walking posture across E15 conditions. (**B**) There were no differences in the scores of gait quality across E15 conditions. (**D**) Offspring exposed to E15 LPS performed better at walking with a correct posture than E15 saline offspring from P18 to P19. ^#^ pairwise comparison; significantly different than saline (*p* < 0.05). *n* = 9–10 rats per sex/group. Error bars represent ±SEM.

**Gait.** The offspring were tested from P5 to P21 to determine their ability to move both forepaws outside of a 15 cm diameter circle within 30 s. There were no significant differences in the acquisition of proper gait as a function of sex [*F*(1, 34) = 0.019, *p* = 0.890], E15 condition [*F*(1, 34) = 0.083, *p* = 0.775], or their interaction [*F*(1, 34) = 1.46, *p* = 0.236]; see Figure 9A.

The repeated measures ANOVA revealed a significant effect of postnatal day [*F*(4.85, 175) = 148, *p* < 0.001, η_p_^2^ = 0.804] on gait, such that the offspring performed better across postnatal age regardless of the E15 condition. There was no significant effect of E15 condition [*F*(1, 36) = 0.017, *p* = 0.899] or the interaction between postnatal day and E15 condition [*F*(16, 576) = 0.966, *p* = 0.493]; see Figure 9B.

**Walking posture.** The offspring were tested from P11 to P21 to determine their ability to walk with their legs positioned under their body and their stomach off the table. There were no significant differences in the acquisition of walking posture as a function of sex [*F*(1, 34) = 0.570, *p* = 0.455], E15 condition [*F*(1, 34) = 2.59, *p* = 0.117], or their interaction [*F*(1, 34) = 0.182, *p* = 0.672]; see Figure 9C.

However, the repeated measures ANOVA revealed a significant effect of postnatal day [*F*(2.99, 108) = 182, *p* < 0.001, η_p_^2^ = 0.835], E15 condition [*F*(1, 36) = 5.80, *p* = 0.021, η_p_^2^ = 0.139], and their interaction [*F*(10, 360) = 4.29, *p* < 0.001, η_p_^2^ = 0.106] on walking posture. Pairwise comparisons revealed that the E15 LPS offspring performed better at walking with a correct posture on P18 (*p* = 0.008) and P19 (*p* = 0.044) than the E15 saline offspring; see Figure 9D.

### 3.3. Maternal Care

**Figure 10:**
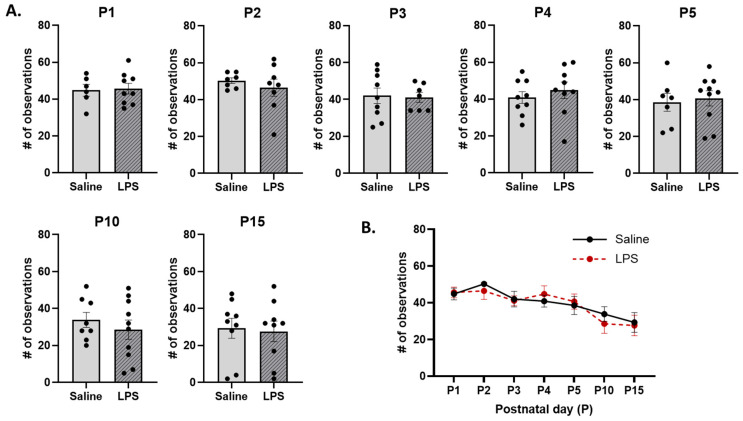
Arched-back nursing (ABN). (**A**) There were no differences in the frequency of ABN across E15 conditions on any postnatal day. (**B**) There were no differences in ABN as a function of E15 condition across postnatal days. However, ABN was significantly lower on P15 than on P2. *n* = 6–10 rats per group. Error bars represent ±SEM.

**Arched-back nursing.** The *t*-tests revealed no significant differences in the frequency of ABN across E15 conditions on each postnatal day [P1: *t*(11.6) = 0.194, *p* = 0.996; P2: *t*(8.53) = 0.781, *p* = 0.982; P3: *t*(13) = 0.171, *p* = 0.996; P4: *t*(14.4) = 0.710, *p* = 0.982; P5: *t*(13) = 0.333, *p* = 0.996; P10: *t*(15.8) = 0.798, *p* = 0.982; P15: *t*(16) = 0.215, *p* = 0.996]; see Figure 10A. Moreover, the mixed-effects analysis revealed a significant difference in the frequency of ABN behaviors across postnatal days [*F*(4.01, 57.5) = 5.80, *p* < 0.001, η_p_^2^ = 0.288]. The pairwise comparison revealed that, regardless of E15 condition, ABN occurred more frequently on P2 than P15 (*p* = 0.032); see Figure 10B. There were no significant effects of E15 condition [*F*(1, 17) = 0.0589, *p* = 0.811] or its interaction with postnatal day [*F*(6, 86) = 0.296, *p* = 0.937].

**Figure 11:**
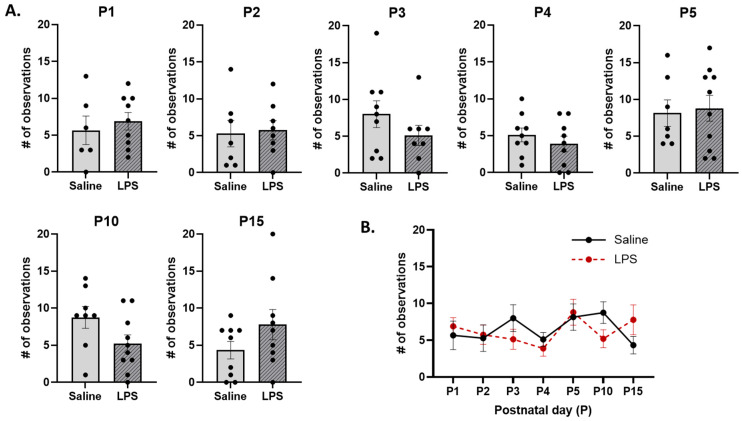
Licking and grooming (LG). (**A**) There were no differences in the frequency of LG between E15 conditions on any postnatal day. (**B**) There were no differences in LG as a function of E15 condition, postnatal day, or their interaction. *n* = 6–10 rats per group. Error bars represent ±SEM.

**Licking and grooming.** The *t*-tests revealed no significant differences in the frequency of LG across E15 conditions on each postnatal day [P1: *t*(13) = 0.574, *p* = 0.924; P2: *t*(13) = 0.213, *p* = 0.961; P3: *t*(15) = 1.24, *p* = 0.736; P4: *t*(16) = 0.871, *p* = 0.867; P5: *t*(15) = 0.255, *p* = 0.961; P10: *t*(16) = 1.88, *p* = 0.434; P15: *t*(16) = 1.47, *p* = 0.651]; see Figure 11A. Furthermore, the mixed-effects analysis revealed no significant difference in the frequency of LG behaviors across postnatal days [*F*(4.16, 60.3) = 1.90, *p* = 0.120,], E15 condition [*F*(1, 17) = 0.0558, *p* = 0.816] or their interaction [*F*(6, 87) = 1.93, *p* = 0.085]; see Figure 11B.

**Figure 12:**
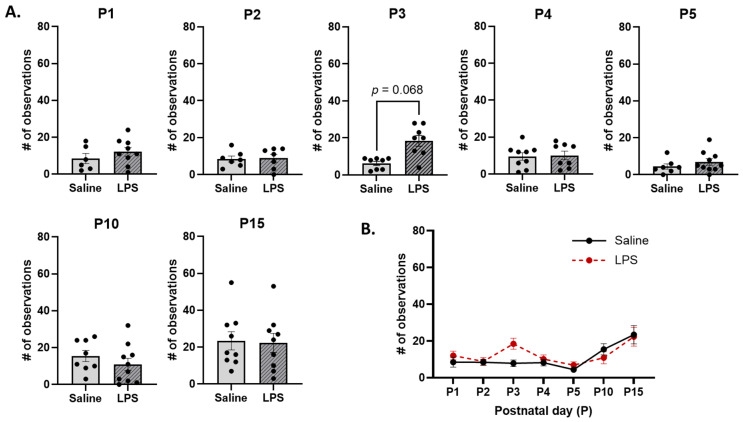
Non-arched-back nursing (non-ABN). (**A**) There were no differences in the frequency of non-ABN across E15 conditions on any postnatal day. (**B**) There were no differences in non-ABN as a function of E15 condition across postnatal days. However, non-ABN was significantly higher on P15 than on P2 and P5. *n* = 6–10 rats per group. Error bars represent ±SEM.

**Non-arched-back nursing.** The *t*-tests revealed no significant differences in the frequency of non-ABN across E15 conditions on each postnatal day [P1: *t*(11.5) = 1.01, *p* = 0.876; P2: *t*(11.1) = 0.131, *p* = 0.984; P3: *t*(11.7) = 3.07, *p* = 0.068; P4: *t*(13.1) = 0.650, *p* = 0.894; P5: *t*(15) = 1.09, *p* = 0.876; P10: *t*(16) = 1.03, *p* = 0.876; P15: *t*(16) = 0.155, *p* = 0.984]; see Figure 12A. Moreover, the mixed-effects analysis revealed a significant difference in the frequency of non-ABN behaviors across postnatal days [*F*(2.56, 43.1) = 6.98, *p* = 0.001, η_p_^2^ = 0.293]. The pairwise comparison revealed that, regardless of E15 condition, non-ABN occurred more frequently on P15 than on P2 (*p* = 0.011) and P5 (*p* = 0.003); see Figure 12B. There were no significant effects of E15 condition [*F*(1, 101) = 1.34, *p* = 0.250] or its interaction with postnatal day [*F*(6, 101) = 1.25, *p* = 0.286].

**Figure 13:**
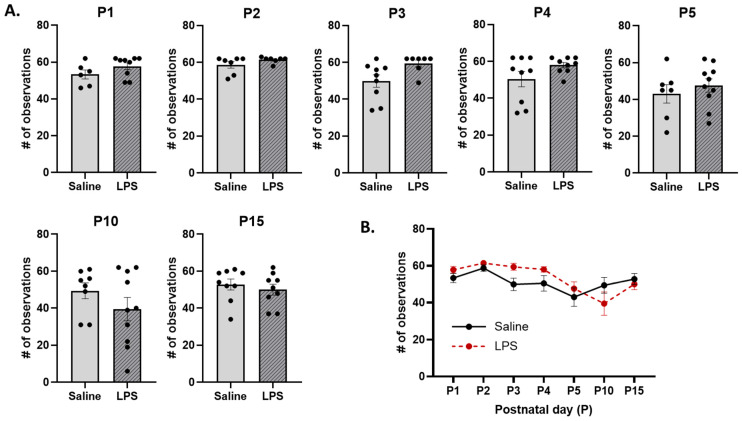
Total nursing. (**A**) There were no differences in the frequency of total nursing across E15 conditions on any postnatal day. (**B**) There were no differences in total nursing behavior as a function of E15 condition across postnatal days. However, total nursing was significantly higher on P2 than on P5. *n* = 6–10 rats per group. Error bars represent ±SEM.

**Total nursing.** The *t*-tests revealed no significant differences in the frequency of total nursing across E15 conditions on each postnatal day [P1: *t*(10.1) = 1.43, *p* = 0.633; P2: *t*(7.41) = 1.45, *p* = 0.633; P3: *t*(12.2) = 2.47, *p* = 0.187; P4: *t*(9.9) = 1.70, *p* = 0.534; P5: *t*(11.9) = 0.745, *p* = 0.720; P10: *t*(15) = 1.30, *p* = 0.633; P15: *t*(16) = 0.663, *p* = 0.720]; see Figure 13A. Moreover, the mixed-effects analysis revealed a significant difference in the frequency of total nursing behaviors across postnatal days [*F*(3.03, 51.4) = 4.71, *p* = 0.005, η_p_^2^ = 0.217]. The pairwise comparison revealed that, regardless of E15 condition, total nursing occurred more frequently on P2 than on P5 (*p* = 0.035); see Figure 13B. There were no significant effects of E15 condition [*F*(1, 102) = 1.40, *p* = 0.239] or its interaction with postnatal day [*F*(6, 102) = 1.76, *p* = 0.115].

## 4. Discussion

The results of these experiments show that MIA with LPS accelerates the acquisition and enhances the performance of numerous developmental reflexes (e.g., forelimb grasping, hindlimb grasping, cliff avoidance, righting with correct posture, walking with correct posture, as described more specifically below) as compared to offspring exposed to prenatal saline. We also found that MIA did not impact the emergence of other developmental reflexes like righting with immature posture, eye opening, and gait. These findings are unlikely to be attributed to changes in maternal care, as we found no differences in the frequency of maternal care behaviors (ABN, LG, non-ABN, and total nursing) by dams toward pups on P1–P5, P10, or P15.

### 4.1. MIA Accelerates Acquisition and Improves Performance of Developmental Reflexes in Neonatal Offspring

There is strong epidemiological evidence to support the idea that individuals with NDDs experience delays in reaching behavioral milestones across development [20,21,22,23,24,25,26,27]. We therefore predicted that MIA would produce delayed acquisition of neonatal developmental reflexes in rat offspring. However, contrary to our hypothesis, we found that MIA with LPS on E15 caused rats to have an earlier acquisition and better performance of various developmental reflexes as compared to saline-exposed offspring. More specifically, we found that E15 LPS offspring were able to forelimb grasp, cliff avoid, and right with a correct posture at an earlier postnatal age than E15 saline offspring. Furthermore, E15 LPS rats performed better at forelimb grasping (from P4 to P6), hindlimb grasping (on P4 and P8), righting with correct posture (from P17 to P18), and walking with correct posture (from P18 to P19) than did E15 saline rats. Notably, we found no differences in the emergence or performance of righting with immature posture, eye opening, nor gait.

Animal models of MIA with LPS and Poly I:C have been reported to cause delays in developmental reflexes such as righting, forelimb grasping, cliff avoidance, and eye opening [30,31,51] and have also been found to produce no differences in these behaviors between groups [32,33,34]. It is possible that some researchers failed to detect differences in reflex acquisition between groups because those reflexes were tested during a more limited timeframe (on or after P9/10) postnatally, although acquisitional delays in righting and eye opening following MIA have also been reported after P9 [30]. Additionally, we found that E15 LPS caused no differences in some of our neonatal reflexes tested (righting with immature posture, eye opening, gait). On the other hand, in line with our current findings, Harvey and Boksa [33] found that MIA with LPS (50 µg/kg) on E15 and E16 caused offspring to have better performance in terms of grip strength (i.e., suspension time while hanging from a rod) and geotaxis (i.e., turning around to move up an inclined plane) abilities. Our model of MIA also enhanced some neonatal reflex behaviors (forelimb/hindlimb grasping, cliff avoidance, righting/walking with mature posture) across development, even without utilizing these more challenging motor tasks (inclined plane and forelimb suspension) to reveal such an effect. In all, the relationship between MIA and neonatal reflex acquisition seems to vary greatly across animal models. Taken together, these findings suggest that MIA does not consistently produce changes across groups in every type of neonatal reflex behavior that can be measured across development. Similarly, both within and across NDD diagnoses in humans, not every person may experience the same symptoms [5]. Thus, the discrepancies in reflex development effects among the current study and the previous literature may reflect natural variability in the consequences of MIA as a risk factor for symptoms of NDDs. This idea of individual variability in the response to MIA, and its interaction with other environmental or genetic components (i.e., mouse vs. rat models, rodent strain, environmental stressors or noise, timing/dose/type of immunogen, etc.), is discussed further below.

Another potential reason for these conflicting results across the current and previous studies may be the timing and dosage of MIA. In both human and rodent studies, there is evidence that the gestational timing and severity of MIA can alter behavioral and neural outcomes in both the mother and the offspring [7,8,44,52,53,54,55]. For instance, lower doses of LPS (100–500 ug/kg) and Poly I:C cause subtle changes in cytokine expression, microglia activation, and neuronal function, whereas larger doses of LPS (over 1 mg/kg) can severely damage white matter, axons, and dendrites [7,8,44,52,55,56]. It is plausible that the lower dose of LPS used in the current study may have affected neonatal reflex development differently than if a larger dose was administered, or if Poly I:C was instead used as the immunogen. Additional studies should be conducted to determine how prenatal exposure to LPS doses between 50 µg/kg and 100 µg/kg, or even doses that are larger or smaller than this range, affect the behavioral outcomes tested in the current study. Researchers should also consider the pathogenic derivation (*E. coli* vs. *Porphyromonas gingivalis*) [57], the serotype, and the preparation (freeze–thaw cycles, pipette/scale calibration, vehicle composition, lot number, etc.) of the LPS products in their studies. For instance, distinct *E. coli* LPS serotypes (of the same dose) can differentially activate inflammatory pathways in myometrium and in neonatal offspring brains [58]. Thus, further research is needed—with a focus on the timing, type, and dosage of the immune challenge—to parse the nuanced neurodevelopmental changes that underlie these differences in developmental reflex behavior across studies.

Additionally, the gestational timing of MIA is important due to the involvement of the neuroimmune system in development. Microglia, the innate immune cells of the brain, are important regulators of neurodevelopment [59]. During early development, microglia populate neural progenitor cell (NPC) proliferation zones and regulate their survival, impacting neurogenesis and gliogenesis [60,61]. Later in development, microglia regulate oligodendrocyte progenitor cells (OPCs) and myelination and are important for the proper maturation of dendritic spines and synapses [60,62,63,64,65]. Due to the role of microglia in both neuroinflammation and typical neurodevelopment, inflammatory challenges such as MIA can compromise microglia function and impact developmental processes differently depending on when the immune response occurs [66,67,68,69]. The effects of this dysregulation may also extend beyond the fetal period and alter offspring neurodevelopment and behavior during the postnatal period [70,71].

One theory as to why our model of MIA may induce earlier acquisition of neonatal reflexes is the stress acceleration hypothesis, whereby early-life adversity—prenatal immune activation being just one example—may cause development to occur on a faster timescale. In conditions of high stress, evolutionary theory suggests that faster development can allow for earlier transition into reproductive stages [72,73]. This could explain the quicker acquisition of motor reflexes across early development seen in the current study. In support of this hypothesis, MIA with Poly I:C has been shown to initiate the early onset of puberty in both male and female offspring [74]. Although beneficial for reproduction, such accelerated development can result in other adverse outcomes such as poor school performance, worse health outcomes, and increased risk of psychiatric disorders later in life [72,73], which are symptoms commonly associated with NDDs [75,76,77]. Thus, even though our current findings do not support the notion that MIA causes behavioral delays associated with NDDs, the data still provide insight into how MIA with LPS impacts reflex development in offspring.

Moreover, our results could be indicative of the individual susceptibility or resilience of the dams and/or fetuses to MIA. Indeed, we found that MIA offspring had significantly different levels of variance in some reflex scores than saline offspring. For instance, homogeneity of variance was violated for forelimb grasping, hindlimb grasping, and righting analyses. LPS offspring had significantly smaller SEMs than the saline group for forelimb grasping, hindlimb grasping, and righting with a correct posture. However, the LPS offspring had a significantly larger SEM for righting with an immature posture than the saline group. Taken together, these results support the idea that MIA may differentially impact neurobiological and behavioral responses across individuals. It is possible that the rats in the LPS group represent those that survived, or were more resilient to, the adverse outcomes associated with E15 MIA exposure (i.e., smaller litter sizes, dams with no pups by P0, etc.). Such resilience to MIA could perhaps impact developmental processes in the surviving offspring and contribute to the observed acceleration in neonatal reflex development. Previous research that found MIA to enhance [33] or delay [31,51] neonatal reflex development reported no group differences in litter sizes or offspring weights; however, they did not mention if other outcomes associated with MIA (e.g., resorption) were examined. One study reported that Poly I:C at E12.5 in mice caused smaller litters and 45.5% of dams to not give birth by P0 [30]. Even though these findings are similar to those in the current model of MIA (E15 LPS reduced litter sizes and caused 44% of dams to not give birth), the researchers showed Poly I:C to cause *delays* in offspring eye opening and righting reflexes [30]. In all, while individual variability in the maternal and fetal consequences of MIA may possibly underlie changes in offspring neurobiological development, its relationship with neonatal reflex behaviors is still unclear.

Many factors—including biological, genetic, and environmental influences—may contribute to one’s individual susceptibility or resilience to the neurobiological and behavioral outcomes of MIA, at both the maternal and fetal levels. For example, MIA can lead to additional pregnancy complications such as pre-eclampsia, which may in turn, itself, contribute to the NDD risk in offspring [78,79]. Environmental factors such as parental age, stress, and dietary deficiencies in the mother or child may also influence NDD susceptibility [5]. Furthermore, genetics can also play a role, as NDDs are known to be highly heritable. Estimates place ASD at a heritability of 80–90% and ADHD at around 75% [80,81,82]. Additionally, some genes associated with NDDs are haploinsufficient, leaving individuals with these genes highly vulnerable to genetic mutations [83]. Together, these studies support the notion that environmental and genetic risk factors likely contribute to individual variability in the immune response to MIA and in the development of NDDs. Although laboratory conditions can control for many variables, it is unsurprising that rodents also show high individual variability in MIA behavioral outcomes, particularly among outbred strains such as Sprague Dawley rats. Future research should further investigate these factors that may impact one’s neurobiological and immune response to MIA and the subsequent risk of NDDs.

Additionally, the current study found that MIA did not affect the acquisition of hindlimb grasping, righting, eye opening, gait, and walking posture (or the quality of righting, cliff avoidance, eye opening, and gait behaviors), which is in line with some previous MIA research described above [32,33,34]. These results likely indicate that the current model of MIA with LPS on E15 simply does not affect offspring performance of these specific behaviors. Another reason for our findings may be due to the sample size, particularly in measuring hindlimb grasping outcomes where significant differences in behavior quality were only detected at P4 and P8. Researchers should consider utilizing larger sample sizes to statistically account for differences in variability between LPS and saline groups. It is also possible that we were unable to detect differences in these reflexes across E15 conditions due to *how* the behaviors were measured in our experiment. Thus, future studies should consider additional methods of assessing the acquisition and performance of behavioral reflexes. The timing of how long it takes a pup to complete a reflex, or more specific measures of the quality of the reflexes performed, can provide more nuanced data that may better reveal developmental differences caused by MIA.

Behaviors other than motor reflexes could also be examined, as individuals with NDDs often experience deficits in communication [84,85] and disruptions to sensory processing [86,87,88]. Therefore, models of MIA should consider utilizing other measures, such as odor preference or recognition learning [89,90,91], tactile stimulation [74,92], and ultrasonic vocalizations [32,89,93], that may reveal additional effects of MIA on early offspring development. Future research should also consider exploring whether reduced maternal weight following MIA is indicative of “maternal toxicity” and if decreased food intake by the dam itself affects the neonatal reflexes separately from the immune response to MIA. Finally, we found no differences in neonatal reflex acquisition or performance between male and female offspring, which suggests our model of MIA does not precipitate sex differences in neonatal behavioral development.

### 4.2. Maternal Care Toward Pups Was Not Affected by MIA

As predicted, we did not detect any significant differences between the E15 conditions in terms of the maternal care behaviors (i.e., ABN, LG, non-ABN, and total nursing) toward the offspring. These findings are in line with previous literature showing that MIA does not affect these maternal care behaviors [32,34,38,39,40]. Since our MIA model with LPS on E15 produces litters of smaller sizes, it could be possible that the litter size influenced the dams’ maternal care behaviors. However, we did not find any significant correlations between the litter size and ABN, LG, nor non-ABN behaviors on P1–P5, P10, and P15 (see Tables S2–S4) which is supported by the literature suggesting that litter size does not affect the frequency of such behaviors [37]. For total nursing behavior, there was a positive correlation with the litter size on P15 (but not on P1–P5 or P10; see Table S5), suggesting that dams with larger litters engaged in more nursing behavior on this postnatal day. This is unlikely to account for the acceleration of neonatal reflex behaviors in the current study, however, as there was no significant relationship with the litter size and ABN or non-ABN at P15.

Even though we did not find any differences between the E15 conditions in terms of ABN or LG—which occur at high levels after birth [37,49]—or in non-ABN, future studies should examine other forms of maternal behavior toward the litter, such as time spent away from the nest, self-grooming, and resting or sleeping. Pup retrieval is another important task that should be considered, as previous research has shown there to be differences in pup retrieval timing as a function of MIA exposure [38,39,40]. The measure (number of observations) of care behaviors used in this study may also have affected our outcomes; quantifying the time spent performing each behavior may possibly reveal more subtle differences in maternal care across conditions.

The maternal care observations in the current study were performed in dams whose pups underwent neonatal developmental reflex testing. It is therefore possible that maternal care behaviors were influenced by the daily separation/handling [94,95] of the dam from pups that underwent testing. If this is true for the current study, it seems that the dams were equally affected across the E15 conditions in their ABN, non-ABN, total nursing, and LG behaviors. Still, future MIA studies should continue to observe and measure maternal care for all the experimental cohorts, as additional factors independent of the study design (e.g., construction, ambient noise, room temperature, etc.) may also differently affect dams across conditions.

Lastly, individual variations in maternal care levels can have profound, long-lasting, and differential effects on male and female offsprings’ neurobiology and behavior [35,96,97] and could contribute to the individual variability seen within a group. We did find a significant positive correlation for LG between P2 and P15 and between P3 and P10 (see Table S3). This could suggest that some dams display higher levels of maternal LG than others at both early and later postnatal days, regardless of their E15 condition. Similarly, the P1 and P5 levels of ABN (see Table S2), the P5 and P15 levels of non-ABN (see Table S4), and the P1 and P3 levels of total nursing (see Table S5), showed significant positive correlations in this study. Champagne and colleagues [37] reported that individual differences in maternal behavior at P8 can reliably be predicted after six consecutive days of observation. Thus, data from additional postnatal days may be needed for us to determine if there are indeed individual variations in the level of maternal care across dams. Future studies should account for and examine how natural variations in maternal care might interact with MIA and contribute to any within-group variability in experimental outcomes.

## 5. Conclusions

Overall, the results of these experiments confirm that MIA with LPS on E15 affects behavioral reflexes in male and female offspring across neonatal development. We predicted that MIA may cause developmental delays in the acquisition of neonatal reflexes and that these effects would be more pronounced in male offspring. Contrary to this hypothesis, we found that MIA-exposed offspring performed reflex behaviors *earlier* and better than saline-exposed offspring, regardless of sex. This is unlikely to be due to changes in maternal care, as we found no significant differences in LG or nursing behaviors (ABN, non-ABN, total nursing) across E15 conditions. Our reflex acquisition behavioral findings could perhaps be attributed to the stress acceleration hypothesis, whereby MIA (an early-life stressor) causes faster development as an evolutionary defense to promote reproduction and survival. In this case, it is thought that MIA may produce such early-life stress via fetal immune dysregulation that persists postnatally. Disruptions in homeostatic immune signaling can interfere with typical neural development, as microglia are in constant communication with neurons and can influence the formation and maintenance of synapses important for reflex behaviors. Future studies should investigate the role of the fetal immune system—by quantifying or perhaps altering the fetal cytokine response following MIA, or by inducing a fetal-specific immune response via intrauterine injection—in postnatal outcomes in offspring. Although MIA is a known risk factor for NDDs and their symptoms, the neurobiological underpinnings of this relationship are still relatively unknown. The current findings thus provide important insight into the effects of prenatal MIA on the ontogeny of behavioral reflex milestones in male and female offspring.

## Data Availability

The original data presented in the study results are openly available in Synapse at SynID: syn64776111, https://www.synapse.org/Synapse:syn64776111.2/datasets/, accessed on 1 February 2025.

## Supplementary Materials

The following supporting information can be downloaded at https://www.mdpi.com/article/10.3390/biom15030347/s1, Table S1: Maternal immune activation model reporting guidelines checklist; Table S2: Arched-Back Nursing (ABN) Correlations; Table S3: Licking and Grooming (LG) Correlations; Table S4: Non-Arched-Back (non-ABN) Nursing Correlations; Table S5: Total Nursing Correlations.

## Abbreviations

The following abbreviations are used in this manuscript:

MIA	Maternal immune activation
NDDs	Neurodevelopmental disorders
LPS	Lipopolysaccharide
Poly I:C	Polyinosinic:polycytidylic acid
ABN	Arched-back nursing
LG	Licking/grooming
Non-ABN	Non-arched-back nursing
