# Supplementary material for: Prenatal Maternal Immune Activation with Lipopolysaccharide Accelerates the Developmental Acquisition of Neonatal Reflexes in Rat Offspring Without Affecting Maternal Care Behaviors
Source: Biomolecules. 2025 Feb 27;15(3):347. doi: 10.3390/biom15030347 (PMC11940702; doi:10.3390/biom15030347)
Supplement: Supplementary file 1 [file biomolecules-15-00347-s001.zip › biomolecules-3482246-supplementary/Supplementary Materials/Supplementary Materials Tables S2-S5.pdf]

Table S2

Arched-Back Nursing (ABN) Correlations

		Litter size	P1	P2	P3	P4	P5	P10	P15
Litter size	Pearson Correlation	--							
	N	19							
P1	Pearson Correlation	-.115	--						
	Sig. (2-tailed)	.684							
	N	15	15						
P2	Pearson Correlation	.011	.312	--					
	Sig. (2-tailed)	.969	.300						
	N	15	13	15					
P3	Pearson Correlation	.285	.501	-.050	--				
	Sig. (2-tailed)	.286	.081	.871					
	N	16	13	13	16				
P4	Pearson Correlation	-.124	.333	-.114	.095	--			
	Sig. (2-tailed)	.623	.226	.685	.727				
	N	18	15	15	16	18			
P5	Pearson Correlation	-.385	.574*	.298	-.104	.254	--		
	Sig. (2-tailed)	.127	.040	.323	.723	.343			
	N	17	13	13	14	16	17		
P10	Pearson Correlation	.136	.299	-.116	.068	.144	-.075	--	
	Sig. (2-tailed)	.590	.300	.693	.810	.582	.784		
	N	18	14	14	15	17	16	18	
P15	Pearson Correlation	.121	-.227	.077	-.197	.053	-.111	-.238	--
	Sig. (2-tailed)	.631	.434	.794	.481	.839	.682	.357	
	N	18	14	14	15	17	16	17	18

* Correlation is significant at the 0.05 level (2-tailed).

Table S3

Licking and Grooming (LG) Correlations

		Litter size	P1	P2	P3	P4	P5	P10	P15
Litter size	Pearson Correlation	--							
	N	19							
P1	Pearson Correlation	-.021	--						
	Sig. (2-tailed)	.940							
	N	15	15						
P2	Pearson Correlation	-.111	.455	--					
	Sig. (2-tailed)	.682	.102						
	N	16	14	16					
P3	Pearson Correlation	.095	.036	.297	--				
	Sig. (2-tailed)	.717	.906	.302					
	N	17	13	14	17				
P4	Pearson Correlation	.193	.337	.172	.438	--			
	Sig. (2-tailed)	.444	.219	.524	.090				
	N	18	15	16	16	18			
P5	Pearson Correlation	.228	.359	.085	.305	.390	--		
	Sig. (2-tailed)	.379	.228	.773	.269	.135			
	N	17	13	14	15	16	17		
P10	Pearson Correlation	.254	-.182	.173	.587*	.311	-.018	--	
	Sig. (2-tailed)	.308	.534	.538	.017	.225	.946		
	N	18	14	15	16	17	16	18	
P15	Pearson Correlation	-.183	.122	.524*	.242	.017	.254	.290	--
	Sig. (2-tailed)	.466	.678	.045	.367	.949	.342	.259	
	N	18	14	15	16	17	16	17	18

* Correlation is significant at the 0.05 level (2-tailed).

Table S4

Non-Arched-Back (non-ABN) Nursing Correlations

		Litter size	P1	P2	P3	P4	P5	P10	P15
Litter size	Pearson Correlation	--							
	N	19							
P1	Pearson Correlation	-.062	--						
	Sig. (2-tailed)	.818							
	N	16	16						
P2	Pearson Correlation	-.030	.470	--					
	Sig. (2-tailed)	.920	.123						
	N	14	12	14					
P3	Pearson Correlation	-.122	.047	.065	--				
	Sig. (2-tailed)	.652	.879	.850					
	N	16	13	11	16				
P4	Pearson Correlation	.446	.030	-.076	.285	--			
	Sig. (2-tailed)	.073	.918	.805	.323				
	N	17	14	13	14	17			
P5	Pearson Correlation	.115	.145	.396	.030	.285	--		
	Sig. (2-tailed)	.660	.621	.202	.920	.304			
	N	17	14	12	14	15	17		
P10	Pearson Correlation	.013	.190	-.436	.026	-.120	-.262	--	
	Sig. (2-tailed)	.958	.499	.136	.924	.658	.326		
	N	18	15	13	16	16	16	18	
P15	Pearson Correlation	.183	-.031	.188	-.311	-.166	.551*	-.293	--
	Sig. (2-tailed)	.468	.912	.537	.260	.539	.027	.253	
	N	18	15	13	15	16	16	17	18

* Correlation is significant at the 0.05 level (2-tailed).

Table S5

Total Nursing Correlations

		Litter size	P1	P2	P3	P4	P5	P10	P15
Litter size	Pearson Correlation	--							
	N	19							
P1	Pearson Correlation	-.226	--						
	Sig. (2-tailed)	.418							
	N	15	15						
P2	Pearson Correlation	-.363	.477	--					
	Sig. (2-tailed)	.202	.117						
	N	14	12	14					
P3	Pearson Correlation	.150	.566*	.454	--				
	Sig. (2-tailed)	.579	.044	.138					
	N	16	13	12	16				
P4	Pearson Correlation	.057	.236	.060	.313	--			
	Sig. (2-tailed)	.823	.397	.838	.239				
	N	18	15	14	16	18			
P5	Pearson Correlation	-.355	-.052	-.051	-.002	-.083	--		
	Sig. (2-tailed)	.162	.866	.875	.994	.761			
	N	17	13	12	14	16	17		
P10	Pearson Correlation	.121	.146	-.243	-.294	.041	-.367	--	
	Sig. (2-tailed)	.631	.619	.423	.287	.876	.162		
	N	18	14	13	15	17	16	18	
P15	Pearson Correlation	.530*	-.179	-.058	.150	-.201	-.324	.413	--
	Sig. (2-tailed)	.024	.540	.850	.592	.438	.220	.100	
	N	18	14	13	15	17	16	17	18

* Correlation is significant at the 0.05 level (2-tailed).
